# Supplementary material for: Structural basis for RNA polymerase II ubiquitylation and inactivation in transcription-coupled repair
Source: Nat Struct Mol Biol. 2024 Feb 5;31(3):536–47. doi: 10.1038/s41594-023-01207-0 (PMC10948364; doi:10.1038/s41594-023-01207-0)
Supplement: Supplementary file 1 — Supplementary Information [file 41594_2023_1207_MOESM1_ESM.pdf]

nature structural & molecular biology

Article

https://doi.org/10.1038/s41594-023-01207-0

Structural basis for RNA polymerase II ubiquitylation and inactivation in transcription-coupled repair

In the format provided by the authors and unedited

Content

- Supplementary Table 1: Primers for cloning
- Supplementary Table 2: Cell lines
- Supplementary Table 3: sgRNAs
- Supplementary Table 4: Plasmids
- Supplementary Table 5: Sequencing primers
- Supplementary Table 6: Antibodies
- References for Supplementary Information

Supplementary Table 1: Primers for cloning

Gene	Sequence	Identifier
ELOF1-WT	5-ACAATTGCTAGCGCCACCATGGGGCGCAGAAAGTC-3	oML#308
	5-AGCTGTGTAAACCGGTAGCTGATTGGCCGCCTCG-3	oML#309
UVSSA-WT	5-CAGAGATCTATGGATCAGAAACTTTCGAAGTTGG-3	oML#741
	5-GCAGAATTCCTAGTTCAGTGCGTAGTTAAACTGG-3	oML#742
UVSSA_C585A-H588A	5-ACCGGCTGAAGGCCCCTTTCGCTGGGAAGA-3	oML#685
	5-TCTTCCCAGCGAAAGGGGCCTTCAGCCGGT-3	oML#686
UVSSA_C567A-C577A	5-AGCACTGGGCCCGTGCCCCGAGGCCAGACGGCCGGCTCGCTGAGCGCCA-3	oML#687
	5-TGGCGCTCAGCGAGCCGGCCGTCTGGCCTCGGGGCACGGGCCCAGTGCT-3	oML#688
UVSSA_Δ661-709 (ΔC)	5-GACGAATTCTTAGTTGGTGAGGCTGGGGTACCTCC-3	oML#743
UVSSA_K679A-R683A	5-AGTCTTCGCCGCAGCAGCTGTGGCAAGGGTAGTGG-3	oML#744
	5-CCACTACCCTTGCCACAGCTGCTGCGGCGAAGACT-3	oML#745
UBCH5B C85K	5- GGCAGCATTAAACTTGATATTCTACGATCACAGTGGTCTCCA-3	
	5- AATATCAAGTTTAATGCTGCCATTACTGTTAATATTTGGATG-3	
CSA-WT	5-CTATCGAGATCTATGCTGGGGTTTTTGT-3	oML#1102
	5-ACTGAGGTCGACTTACTTGTACAGCTCGTCCA-3	oML#1103
CSA-Y334A	5-AGGGACATGCTAAAACTGTTGA-3	oML#635
	5-TCAACAGTTTTAGCATGTCCCT-3	oML#636

Supplementary Table 2: Cell lines

Cell lines	Source
HEK293T	ATCC CRL-3216
Hi5 / Tni insect cells	Expression systems, 94-002F
RPE1-iCas9	[1]
RPE1-iCas9 CSA-KO (3-8)	[1]
RPE1-iCas9 CSA-KO (3-8) + CSA^{WT}-GFP	This study
RPE1-iCas9 CSA-KO (3-8) + CSA^{Y334A}-GFP	This study
RPE1-iCas9 CSB-KO (1-15)	[1]
RPE1-iCas9 ELOF1-KO (2-16)	[1]
RPE1-iCas9 ELOF1-KO (2-16) + GFP- ELOF1$^{E55A-E79A}$	This study
RPE1-iCas9 ELOF1-KO (2-16) + GFP- ELOF1$^{N30A-H31A-E32A}$ (Δdock)	This study
RPE1-iCas9 ELOF1-KO (2-16) + GFP-ELOF1 $^{N30A-H31A-E32A-E55A-E79A}$	This study
RPE1-iCas9 ELOF1-KO (2-16) + GFP-ELOF1^{WT}	This study
RPE1-iCas9 UVSSA-KO (3-9)	[1]
RPE1-iCas9 UVSSA-KO (3-9) + GFP-UVSSA$^{C567A-C577A}$ (ΔZnF2)	This study
RPE1-iCas9 UVSSA-KO (3-9) + GFP-UVSSA$^{C567A-C577A-C585A-H588A}$ (ΔZnF4)	This study
RPE1-iCas9 UVSSA-KO (3-9) + GFP-UVSSA$^{K679A-R683A}$	This study
RPE1-iCas9 UVSSA-KO (3-9) + GFP-UVSSA^{WT}	This study
RPE1-iCas9 UVSSA-KO (3-9) + GFP-UVSSA$^{\Delta 667-699}$ (ΔC)	This study
Sf21 insect cells	Expression systems, 94-003F
Sf9 insect cells	ThermoFisher, 12659017
U2OS (FRT)	Gift from Daniel Durocher (Toronto, Ontario)
U2OS (FRT) UVSSA-KO (1-8) + GFP-UVSSA^{WT}-3	[2]

Supplementary Table 3: sgRNAs

sgRNA	Sequence	Gene_ID	Exon_ID	Exon
CSB_1	CTCATCGGATCATTCTGTCT	ENSG00000225830	ENSE00002514316	10
CSA_3	GGAGAGCAGAGTCAACACGG	ENSG00000049167	ENSE00001762056	1
ELOF1_2	CGAGAAATCCTGTGATGTGA	ENSG00000130165	ENSE00003469475	1
UVSSA_3	GCCGGCTGTGTGCTCGTGGA	ENSG00000163945	ENSE00001713804	6

Supplementary Table 4: Plasmids

Plasmid	Origin	Identifier
pX458	Addgene #48138	pML#006
pX458-(Cas9-2A-GFP)-sgCSB-1	This study	pML#003
pX458-(Cas9-2A-GFP)-sgCSA-3	This study	pML#219
pX458-(Cas9-2A-GFP)-sgELOF1-2	This study	pML#186
pX458-(Cas9-2A-GFP)-sgUVSSA_3	This study	pML#220
PKG-EGFP-C1-IRES-puro	This study	pML#244
PKG-EGFP-N1-IRES-puro	This study	pML#245
PGK-ELOF1-GFP-IRES-puro	This study	pML#273
PGK-ELOF1(N30A-H31A-E32A)-GFP-IRES-puro (Δdock)	This study	pML#274
PGK-ELOF1(E55A-E79G)-GFP-IRES-puro	This study	pML#275
PGK-ELOF1(N30A-H31A-E32A-E55A-E79G)-GFP-IRES-puro (5A)	This study	pML#276
PGK-GFP-UVSSA-IRES-puro-5aa-Linker	This study	pML#313
PGK-GFP-UVSSA-C567A-C577A-IRES-puro (ZnF2)	This study	pML#314
PGK-GFP-UVSSA-C567A-C577A-C585A-H588A-IRES-puro (ZnF4)	This study	pML#315
PGK-GFP-UVSSA-Δ661-709-IRES-puro (ΔC)	This study	pML#316
PGK-GFP-UVSSA-K679A-R683A-IRES-puro	This study	pML#317
pLenti-PGK-GFP-puro	This study	pML#030
pLenti-PGK-CSA-GFP-puro	This study	pML#445
pLenti-PGK-CSA-Y334A-GFP-puro	This study	pML#446
pSMT3-His6-SMT3-UBCH5B-C85K	This study	
pET30a-ubiquitin	[3]	
pFASTBac-His6-UBA1	[3]	

Supplementary Table 5: Sequencing primers

Gene	Sequence	Identifier
UVSSA	5-ACCCAGAGGTACACAGAGATTG-3	oML#090_ sgUVSSA1-1_ FW
	5-GCTCTTAGAAGTGTCCCTGTG-3	oML#091_ sgUVSSA1-1_ RV
	5-ATCAGGAGGCTGAGGCGGCTG-3	oML#076_ sgUVSSA1-2_ FW
	5-AGGAGCCTACCCGGGAGCCGGG-3	oML#077_ sgUVSSA1-2_ RV
ELOF1	5-ATGTTGCCCAGGCTGGTATC-3	oML#320_sgELOF1-2_Seq_FW1
	5-TCCTCTGTGTCGCTACTGATTG-3	oML#321_sgELOF1-2_Seq_RV1
	5-GATCACAGGTGTGAGCCAC-3	oML#328_sgELOF1-2_Seq_FW2
	5-CACTTAGGTCAAGGGCGATC-3	oML#329_sgELOF1-2_Seq_RV2
	5-AAGAAGATGACAGGCACCCTC-3	oML#322_sgELOF1-3_Seq_FW1
	5-CGGGAAGTCCAGTTGAGATG-3	oML#323_sgELOF1-3_Seq_RV1
	5-TGAAGGCGTCATCACCCAC-3	oML#330_sgELOF1-3_Seq_FW2
	5-GCTTTCGGAGCCAAGTGAG-3	oML#331_sgELOF1-3_Seq_RV2
CSA	5-ACTGACCTCGCAATCACTGAC-3	oML#444_sgCSA-3_ FW1
	5-CAAGTACACAAGGCTCTTCCTC-3	oML#445_sgCSA-3_RV1
	5-TTGGTCCGTGCCCACGTG-3	oML#446_sgCSA-3_ FW2
	5-GTCCTCTGCCTTTAATAGGCTG-3	oML#447_sgCSA-3_RV2

Supplementary Table 6: Antibodies

Antibody	Host	Company (reference)	Use	Identifier
CSA/ERCC8	Mouse	Santa Cruz, #sc-376981 (D2)	WB: 1:500	aML#025
CSA/ERCC8	Rabbit	Abcam, #137033 (EPR9237)	WB: 1:500	aML#028
CSB/ERCC6	Rabbit	Santa Cruz, #sc-25370 (H-300)	WB: 1:300	aML#003
CSB/ERCC6	Rabbit	Bethyl Laboratories, #A301-345A	WB: 1:600	aML#187
GFP	Mouse	Roche, #11814460001 (7.1 and 13.1)	WB: 1:1000	aML#011
GFP	Rabbit	Abcam, #ab290	WB: 1:1000	aML#044
Mouse Alexa 555	Goat	Thermo fisher Scientific, A-21424	IF: 1:1000	aML#015
Mouse Alexa 647	Goat	Thermo fisher Scientific, A-21235	IF: 1:1000	aML#017
Mouse IgG (H+L) CF770	Goat	Biotium, VWR #20077	WB: 1:10000	aML#009
p62/GTF2H1	Mouse	Santa Cruz, #sc-48431 (G10)	WB: 1:500	aML#099
p89/XPB/ERCC3	Mouse	Millipore, #MABE1123	WB: 1:2000	aML#101
phospho-H2A.X Ser139	Mouse	Merck, #05-636 (JBW301)	IF: 1:1000	aML#161
Pol II-S2	Rabbit	Abcam, #ab5095	WB: 1:1000	aML#024
Rabbit IgG (H+L) CF680	Goat	Biotium, VWR #20067	WB: 1:10000	aML#010
RBX1	Rabbit	Cell Signaling, 11922S	WB: 1:6000	aML#155
RPB1 (fluorescently labelled 8WG16)	Mouse	Cramer lab in-house purified	WB: 1:1000	
Ubiquitin (FK2)	Mouse	ENZO Life Sciences, BML-PW8810-0500	WB: 1:1000	aML#102
Ubiquitin (P4D1)	Mouse	Cell Signaling, mAb#3936	WB: 1:1000	aML#192
αTubulin	Mouse	Sigma, #T6199 (DM1A)	WB: 1:1000	aML#008

References

1. van der Weegen, Y. et al. ELOF1 is a transcription-coupled DNA repair factor that directs RNA polymerase II ubiquitylation. *Nat Cell Biol* **23**, 595-607 (2021).
2. van der Weegen, Y. et al. The cooperative action of CSB, CSA, and UVSSA target TFIIH to DNA damage-stalled RNA polymerase II. *Nat Commun* **11**, 2104 (2020).
3. Wickliffe, K.E., Lorenz, S., Wemmer, D.E., Kuriyan, J. & Rape, M. The mechanism of linkage-specific ubiquitin chain elongation by a single-subunit E2. *Cell* **144**, 769-81 (2011).
