# Supplementary material for: Structural basis for RNA polymerase II ubiquitylation and inactivation in transcription-coupled repair
Source: Nat Struct Mol Biol. 2024 Feb 5;31(3):536–47. doi: 10.1038/s41594-023-01207-0 (PMC10948364; doi:10.1038/s41594-023-01207-0)
Supplement: Supplementary file 2 — Reporting Summary [file 41594_2023_1207_MOESM2_ESM.pdf]

nature research

Corresponding author(s): Martijn Luijsterburg
Last updated by author(s): 30 November 2023

Reporting Summary

Nature Research wishes to improve the reproducibility of the work that we publish. This form provides structure for consistency and transparency in reporting. For further information on Nature Research policies, see our Editorial Policies and the Editorial Policy Checklist.

Statistics

For all statistical analyses, confirm that the following items are present in the figure legend, table legend, main text, or Methods section.

n/a	Confirmed	
☐	☒	The exact sample size (*n*) for each experimental group/condition, given as a discrete number and unit of measurement
☐	☒	A statement on whether measurements were taken from distinct samples or whether the same sample was measured repeatedly
☒	☐	The statistical test(s) used AND whether they are one- or two-sided<br>*Only common tests should be described solely by name; describe more complex techniques in the Methods section.*
☒	☐	A description of all covariates tested
☒	☐	A description of any assumptions or corrections, such as tests of normality and adjustment for multiple comparisons
☐	☒	A full description of the statistical parameters including central tendency (e.g. means) or other basic estimates (e.g. regression coefficient) AND variation (e.g. standard deviation) or associated estimates of uncertainty (e.g. confidence intervals)
☒	☐	For null hypothesis testing, the test statistic (e.g. *F*, *t*, *r*) with confidence intervals, effect sizes, degrees of freedom and *P* value noted<br>*Give P values as exact values whenever suitable.*
☒	☐	For Bayesian analysis, information on the choice of priors and Markov chain Monte Carlo settings
☒	☐	For hierarchical and complex designs, identification of the appropriate level for tests and full reporting of outcomes
☒	☐	Estimates of effect sizes (e.g. Cohen's *d*, Pearson's *r*), indicating how they were calculated

Our web collection on statistics for biologists contains articles on many of the points above.

Software and code

Policy information about availability of computer code

Data collection	Serial EM 3.8 beta 8; pLink (v. 1.23). Microscopy images were acquired using a Zeiss AxioImager M2 or D2 widefield fluorescence microscope and ZEN 2012 software (blue edition, version 1.1.0.0). Western blot images were acquired using a Odyssey CLx with Image studio lite software (v5.2).
Data analysis	RELION 3.0, UCSF Chimera 1.13, Coot 0.9, Warp v1.0.7, PHENIX 1.18, cryoSPARC 2.14.2, Prism v8.4.2, Molprobity 4.5.1, XlinkAnalyzer version 1.1. Microscopy images were analyzed in Image J (1.47v-1.48v). Graphs were plotted and analyzed using Graphpad Prism 8 (v8.4.2), Microsoft Excel 365, PlotsOfData webtool, and Adobe Illustrator 2021.

For manuscripts utilizing custom algorithms or software that are central to the research but not yet described in published literature, software must be made available to editors and reviewers. We strongly encourage code deposition in a community repository (e.g. GitHub). See the Nature Research guidelines for submitting code & software for further information.

Data

Policy information about availability of data

All manuscripts must include a data availability statement. This statement should provide the following information, where applicable:

- Accession codes, unique identifiers, or web links for publicly available datasets
- A list of figures that have associated raw data
- A description of any restrictions on data availability

The electron density reconstructions and structure coordinates were deposited to the Electron Microscopy Database (EMDB) and to the PDB under the following accession codes: EMD-15825 and PDB 8B3D for the Pol II-TCR-ELOF1 structure, EMD-15829 and PDB 8B3I for the CNRL4CSA-E2-Ub structure and EMD-15827 and PDB 8B3G for the CNRL4CSA-E2-Ub-UVSSA structure. The crosslinking mass spectrometry data have been deposited to the ProteomeXchange Consortium via PRIDE with the dataset identifier PXD042388.

Following structures were used for model building or figure making: Pol II-CSB-CSA-DDB1-UVSSA structure (PDB code 7OO3), NEDD8-CUL1-RBX1 N98R-SKP1-monomeric b-TRCP1dD-IkBa-UB~UBE2D2 (PDB code 6TTU) and RNA polymerase II-TFIIS complex (1PQV).

Field-specific reporting

Please select the one below that is the best fit for your research. If you are not sure, read the appropriate sections before making your selection.

☒ Life sciences ☐ Behavioural & social sciences ☐ Ecological, evolutionary & environmental sciences

For a reference copy of the document with all sections, see nature.com/documents/nr-reporting-summary-flat.pdf

Life sciences study design

All studies must disclose on these points even when the disclosure is negative.

Sample size	No statistical methods were used to predetermine sample size. Sample sizes were chosen for the different experimental approaches based on the technical difficulty and throughput of the individual assays, the chosen sample sizes are consistent with previous publications. All biochemical and cell culture experiments were replicated two or more times. Structural data was collected on five independently prepared samples.
Data exclusions	No data were excluded from the analyses.
Replication	All attempts at replication were successful, at least two repetitions for biochemical assays were performed. Cryo-EM single particle analysis inherently relies on averaging over a large number of independent observations. For cell culture approaches, the number of replicate experiments are indicated in the figure legends of the manuscript. At least two replicates were performed for each individual approach. Effects of knock-out of proteins of interest were confirmed by rescue experiments in at least three independent experiments.
Randomization	There was no allocation of test subjects for any experiments, thus randomization was not applicable to our study
Blinding	Data analyses were performed by unbiased software programs/algorithms blinding was therefore not applicable to our study

Reporting for specific materials, systems and methods

We require information from authors about some types of materials, experimental systems and methods used in many studies. Here, indicate whether each material, system or method listed is relevant to your study. If you are not sure if a list item applies to your research, read the appropriate section before selecting a response.

Materials & experimental systems

n/a	Involved in the study	
☐	☒	Antibodies
☐	☒	Eukaryotic cell lines
☒	☐	Palaeontology and archaeology
☒	☐	Animals and other organisms
☒	☐	Human research participants
☒	☐	Clinical data
☒	☐	Dual use research of concern

Methods

n/a	Involved in the study	
☒	☐	ChIP-seq
☒	☐	Flow cytometry
☒	☐	MRI-based neuroimaging

Antibodies

Antibodies used

CSA/ERCC8 Mouse Santa Cruz, #sc-376981 (D2) WB: 1:500 aML#025
CSA/ERCC8 Rabbit Abcam, #137033 (EPR9237) WB: 1:500 aML#028
CSB/ERCC6 Rabbit Santa Cruz, #sc-25370 (H-300) WB: 1:300 aML#003
CSB/ERCC6 Rabbit Bethyl Laboratories, #A301-345A WB: 1:600 aML#187
GFP Mouse Roche, #11814460001 (7.1 and 13.1) WB: 1:1000 aML#011
GFP Rabbit Abcam, #ab290 WB: 1:1000 aML#044
Mouse Alexa 555 Goat Thermo fisher Scientific, A-21424 IF: 1:1000 aML#015
Mouse Alexa 647 Goat Thermo fisher Scientific, A-21235 IF: 1:1000 aML#017
Mouse IgG (H+L) CF770 Goat Biotium, VWR #20077 WB: 1:10000 aML#009
p62/GTF2H1 Mouse Santa Cruz, #sc-48431 (G10) WB: 1:500 aML#099
p89/XPB/ERCC3 Mouse Millipore, #MABE1123 WB: 1:2000 aML#101
phospho-H2A.X Ser139 Mouse Merck, #05-636 (JBW301) IF: 1:1000 aML#161
Pol II-S2 Rabbit Abcam, #ab5095 WB: 1:1000 aML#024
Rabbit IgG (H+L) CF680 Goat Biotium, VWR #20067 WB: 1:10000 aML#010
RBX1 Rabbit Cell Signaling, 11922S WB: 1:6000 aML#155
RPB1 (fluorescently labelled 8WG16) Mouse Cramer lab in-house purified WB: 1:1000

	Ubiquitin (FK2) Mouse ENZO Life Sciences, BML-PW8810-0500 WB: 1:1000 aML#102<br>Ubiquitin (P4D1) Mouse Cell Signaling, , mAb#3936 WB: 1:1000 aML#192<br>αTubulin Mouse Sigma, #T6199 (DM1A) WB: 1:1000 aML#008
Validation	The following antibodies were validated in knockout cells and Co-IP experiments:<br><br>CSA/ERCC8, Mouse, Santa Cruz #sc-376981 (D2), WB: 1:500, aML#025<br>CSA/ERCC8, Rabbit, Abcam #137033 (EPR9237), WB: 1:500, aML#028<br>CSB/ERCC6, Rabbit, Santa Cruz #sc-25370 (H-300) WB: 1:300 aML#003<br>CSB/ERCC6, Rabbit, Bethyl Laboratories #A301-345A, WB: 1:600 ,aML#187<br><br>The following antibodies were validated in Co-IP experiments:<br><br>GFP, Mouse, Roche #11814460001 (7.1 and 13.1), WB: 1:1000, aML#011<br>p62/GTF2H1, Mouse, Santa Cruz #sc-48431 (G10), WB: 1:500, aML#099<br>p89/XPB/ERCC, 3Mouse, Millipore #MABE1123, WB: 1:2000, aML#101<br>RNAPII-S2, Rabbit, Abcam #ab5095, WB: 1:1000, aML#024<br><br>This antibody was validated by western blot of cells expressing GFP tagged proteins:<br>GFP, Rabbit, Abcam #ab290, WB: 1:1000, aML#044<br><br>This antibody was validated in fluorescence microscopy experiments:<br>phospho-H2A.X Ser139, Mouse, Merck #05-636 (JBW301), IF: 1:1000, aML#161<br><br>This antibody is a commonly used loading control:<br>Tubulin, Mouse, Sigma #T6199 (DM1A), WB: 1:1000, aML#008<br><br>This antibody is a commonly used ubiquitin antibody:<br>Ubiquitin (P4D1), Mouse, Cell Signaling, WB: 1:1000, aML#192

Eukaryotic cell lines

Policy information about cell lines

Cell line source(s)	Sf9 insect cells (ThermoFisher, 12659017) were cultured in Sf-9000TM III SFM medium (Thermo Fisher Scientific)<br>Hi5 (Expression systems, 94-002F) were cultured in ESF921 medium (Expression Systems, 96-001-01)<br>Sf21 cells (Expression systems, 94-003F) were cultured in ESF921 medium (Expression Systems, 96-001-01)<br>HEK293T (ATCC CRL-3216)<br>RPE1-iCas9 (van der Weegen, et al. 2021)<br>RPE1-iCas9 CSB-KO (1-15) (van der Weegen, et al. 2021)<br>RPE1-iCas9 ELOF1-KO (2-16) (van der Weegen, et al. 2021)<br>RPE1-iCas9 ELOF1-KO (2-16) + GFP-ELOF1-WT, This study<br>RPE1-iCas9 ELOF1-KO (2-16) + GFP- ELOF1-N30A-H31A-E32A (Δdock), This study<br>RPE1-iCas9 ELOF1-KO (2-16) + GFP- ELOF1-E55A-E79A, This study<br>RPE1-iCas9 ELOF1-KO (2-16) + GFP-ELOF1-N30A-H31A-E32A-E55A-E79A, This study<br>RPE1-iCas9 UVSSA-KO (3-9) (van der Weegen, et al. 2021)<br>RPE1-iCas9 UVSSA-KO (3-9) + GFP-UVSSA-WT, This study<br>RPE1-iCas9 UVSSA-KO (3-9) + GFP-UVSSA-Δ667-699 (ΔC), This study<br>RPE1-iCas9 UVSSA-KO (3-9) + GFP-UVSSA-K679A-R683A,This study<br>RPE1-iCas9 UVSSA-KO (3-9) + GFP-UVSSA-C567A-C577A (ΔZnF2), This study<br>RPE1-iCas9 UVSSA-KO (3-9) + GFP-UVSSA-C567A-C577A-C585A-H588A (ΔZnF4), This study<br>RPE1-iCas9 CSA-KO (3-8) (van der Weegen, et al. 2021)<br>RPE1-iCas9 CSA-KO (3-8) + CSA-WT-GFP, This study<br>RPE1-iCas9 CSA-KO (3-8) + CSA-Y334A-GFP, This study<br>U2OS (FRT) UVSSA-KO (1-8) + GFP-UVSSAWT-3 (van der Weegen, et al. 2020)<br>U2OS (FRT): Gift from Daniel Durocher (Toronto, Ontario,)
Authentication	None of the cell lines were authenticated.
Mycoplasma contamination	Hi5, Sf9, and Sf21 cell lines were not tested for mycoplasma contamination. All RPE1-iCas9, U2OS (FRT) and HEK293T cell lines were regularly tested for mycoplasma contamination and were negative.
Commonly misidentified lines<br>(See ICLAC register)	No commonly misidentified cell lines were used.
