# Supplementary figures and images for: Structural basis for RNA polymerase II ubiquitylation and inactivation in transcription-coupled repair
Source: Nat Struct Mol Biol. 2024 Feb 5;31(3):536–47. doi: 10.1038/s41594-023-01207-0 (PMC10948364; doi:10.1038/s41594-023-01207-0)

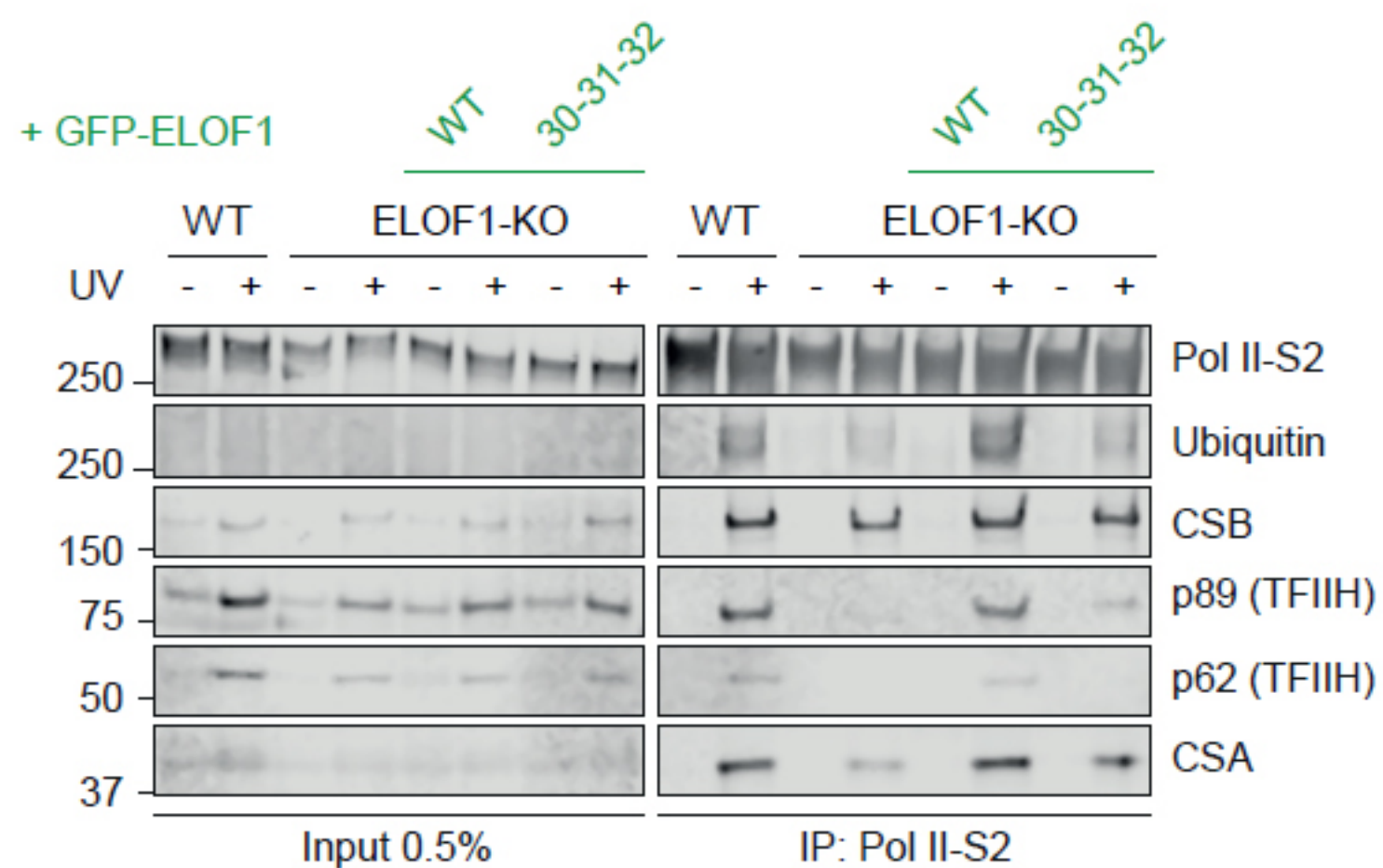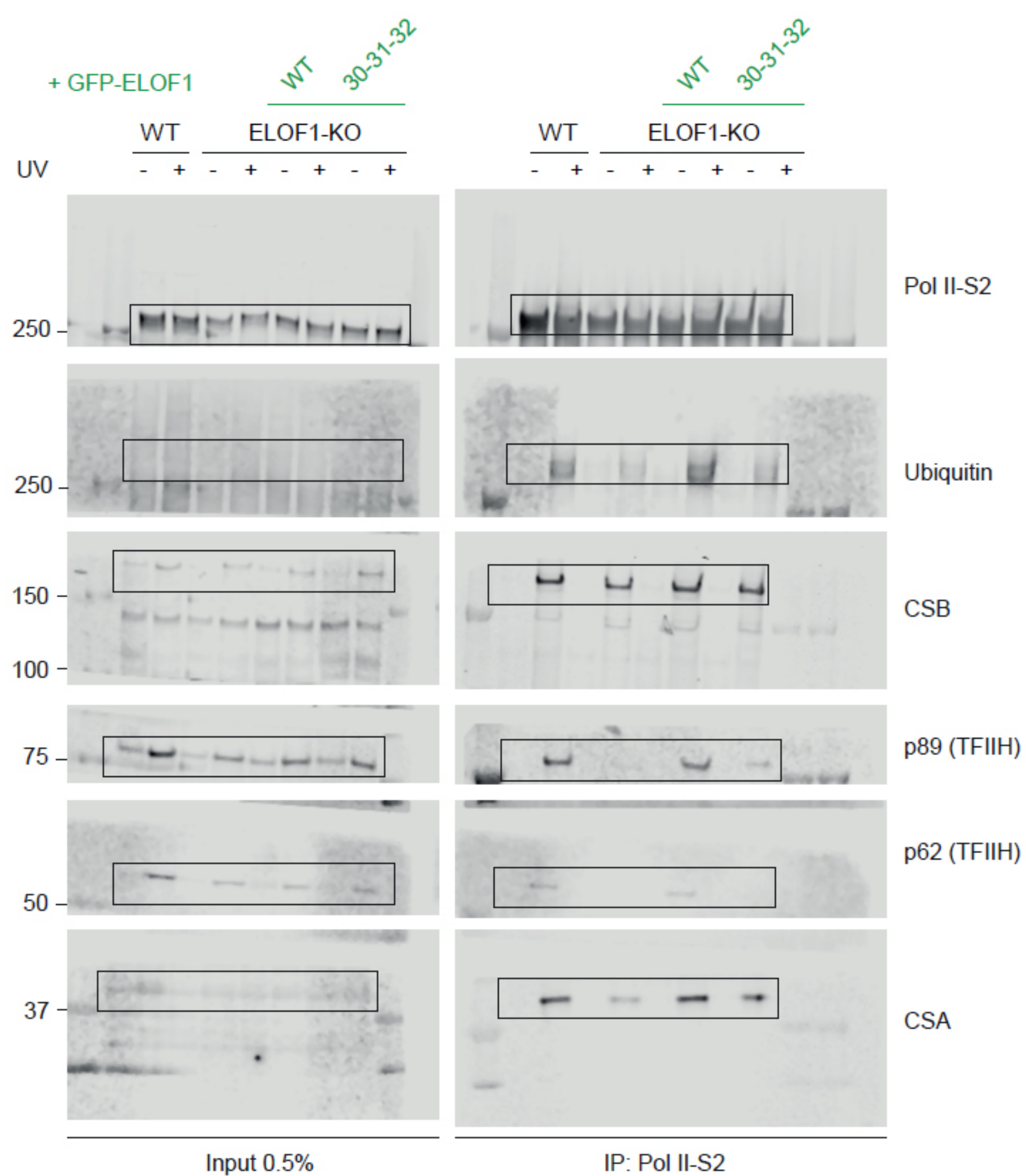

Supplement: Supplementary file 8 — Unprocessed western blots. [file 41594_2023_1207_MOESM8_ESM.pdf]

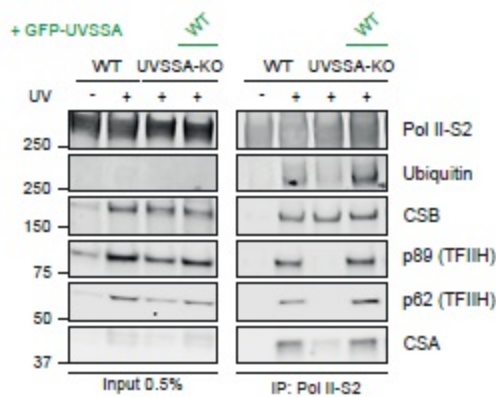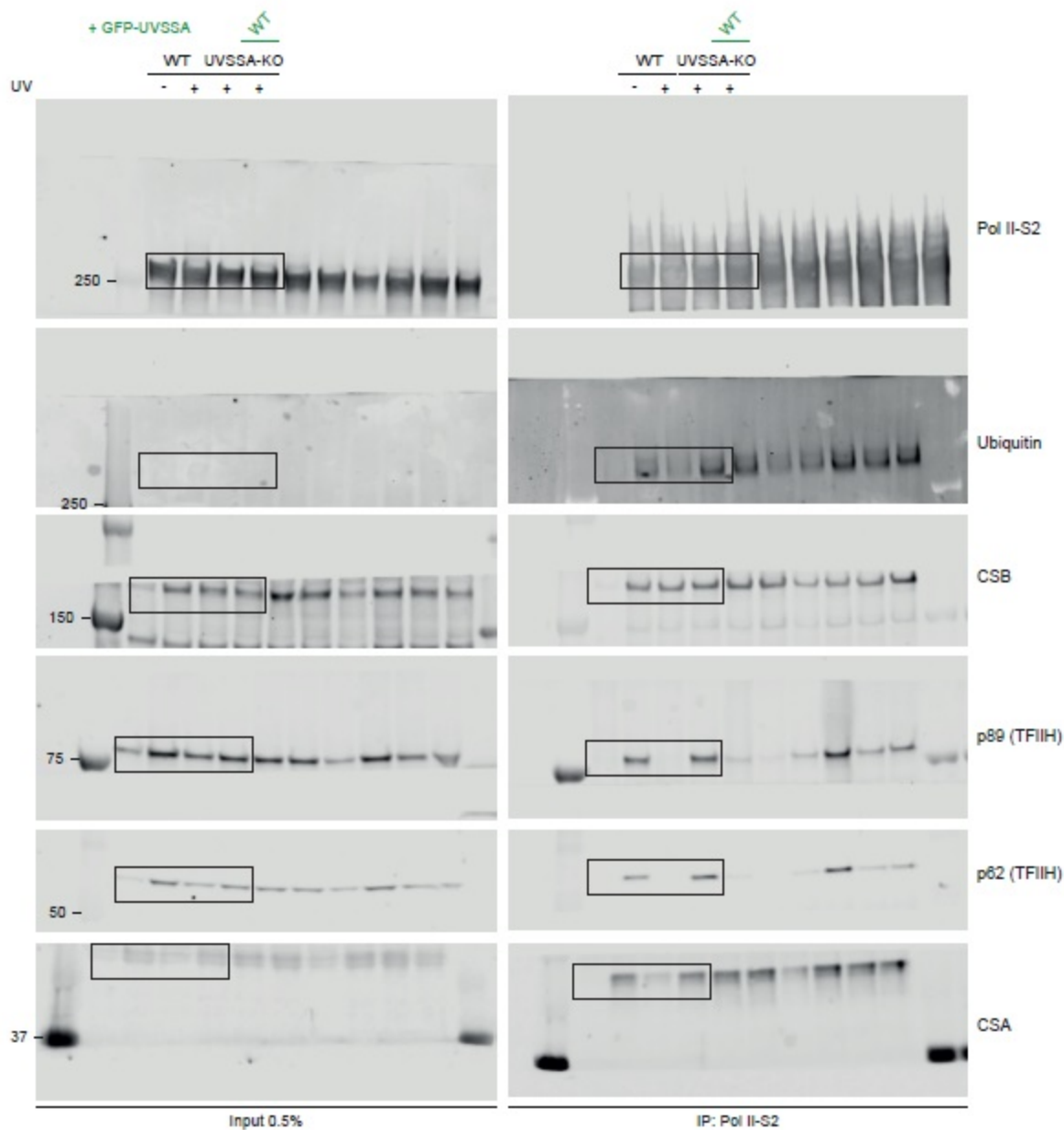

In vitro ubiquitylation  
Pol II-ELOF1-CSB-CRL4<sup>CSA</sup>

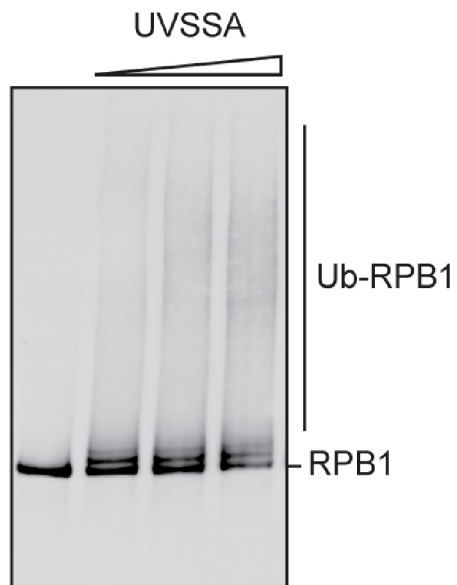

250

180

130

95

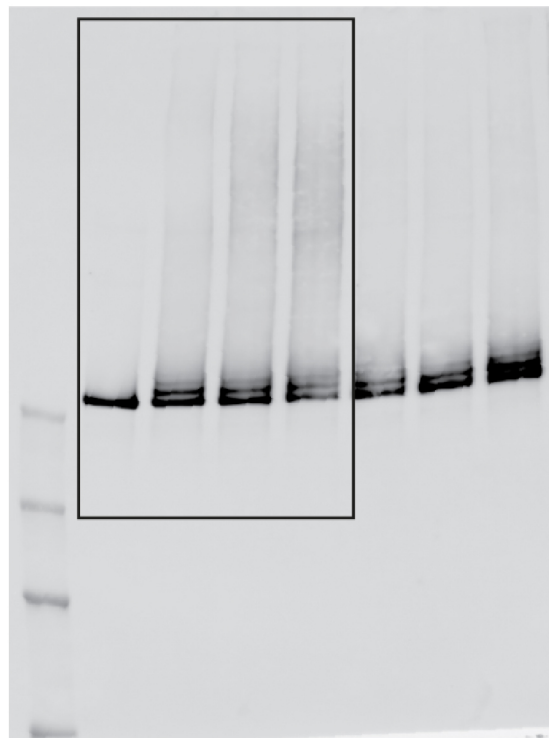

Supplement: Supplementary file 10 — Unprocessed western blots. [file 41594_2023_1207_MOESM10_ESM.pdf]

# Endo PolI-S2 IP with NEM

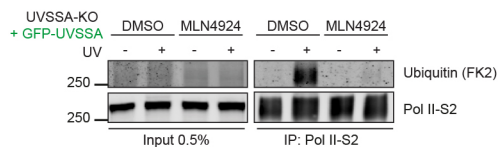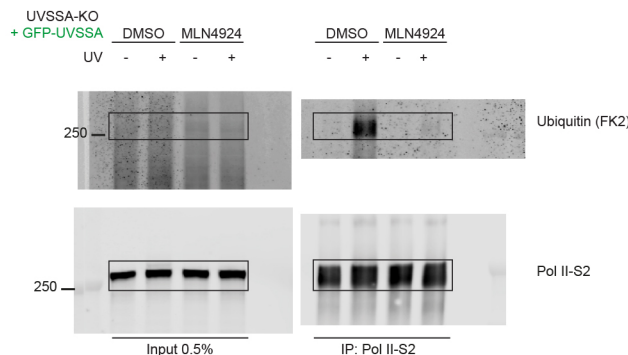

# Endo PolI-S2 IP without NEM

## EndoRNAPII IP without NEM

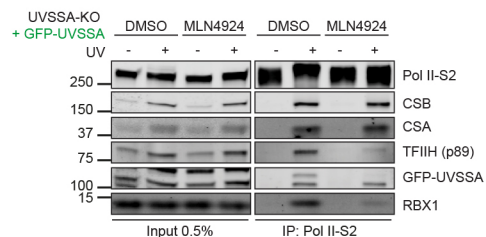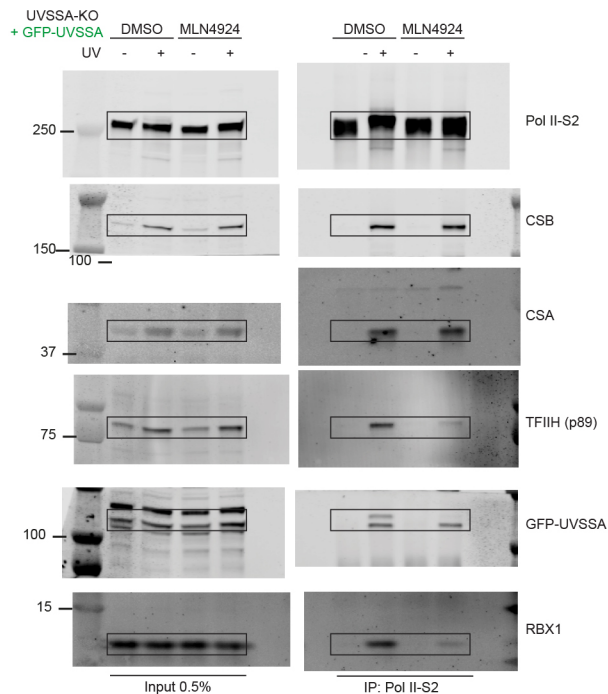

Supplement: Supplementary file 11 — Unprocessed western blots. [file 41594_2023_1207_MOESM11_ESM.pdf]

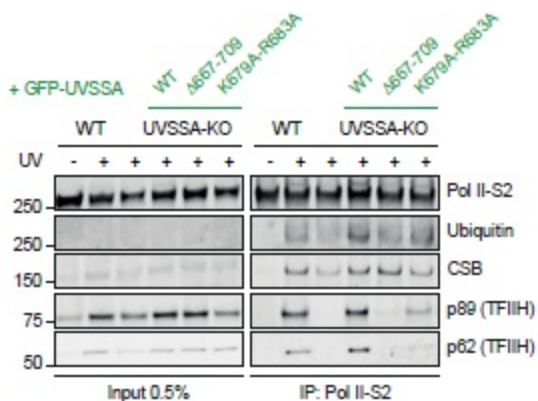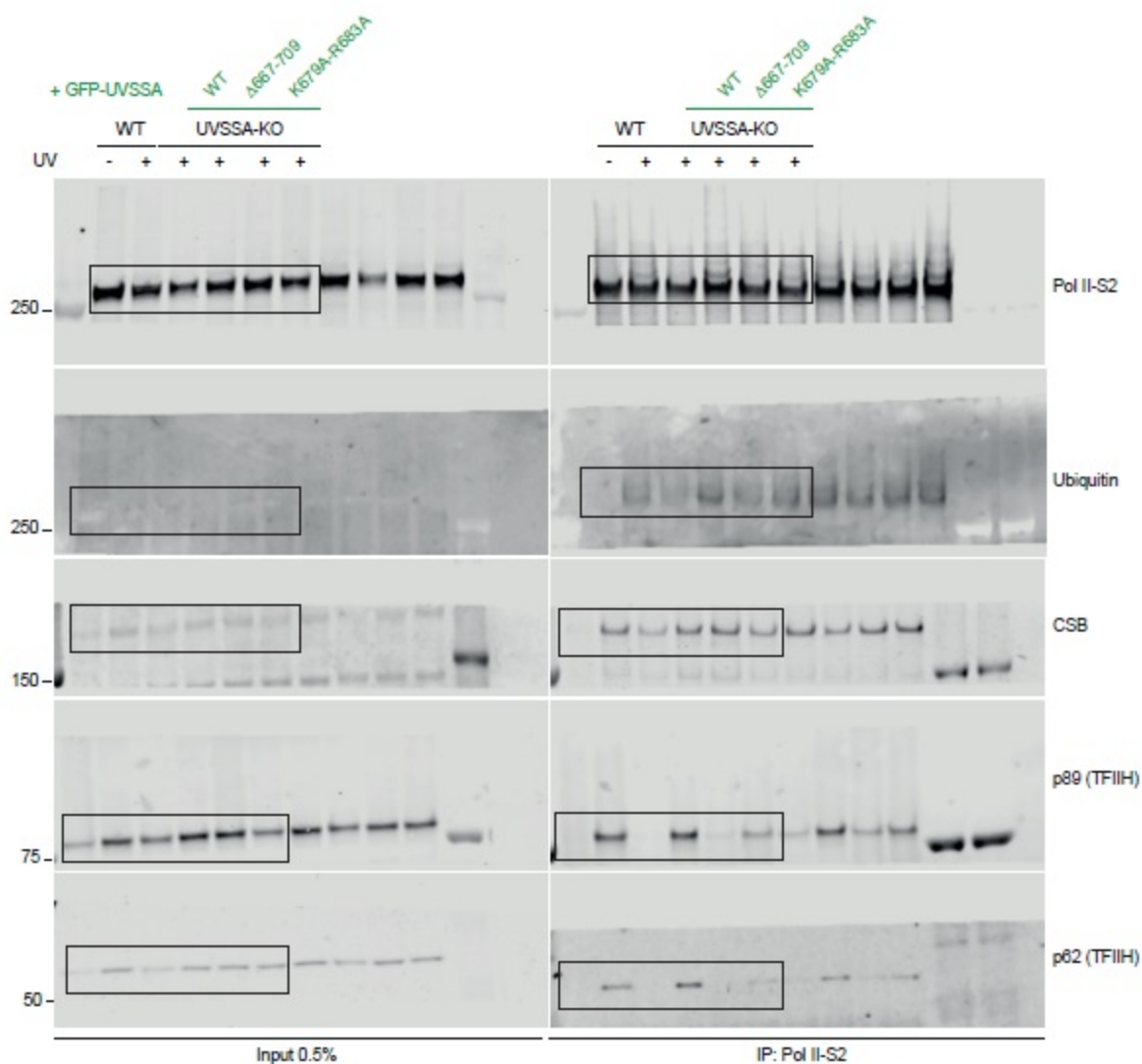

Supplement: Supplementary file 13 — Unprocessed western blots. [file 41594_2023_1207_MOESM13_ESM.pdf]

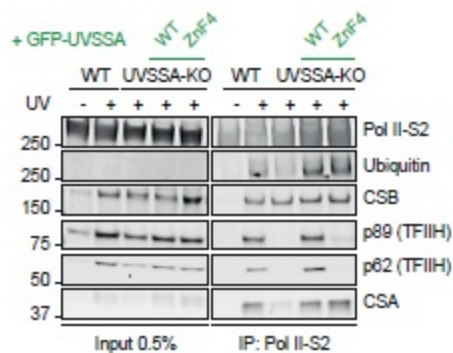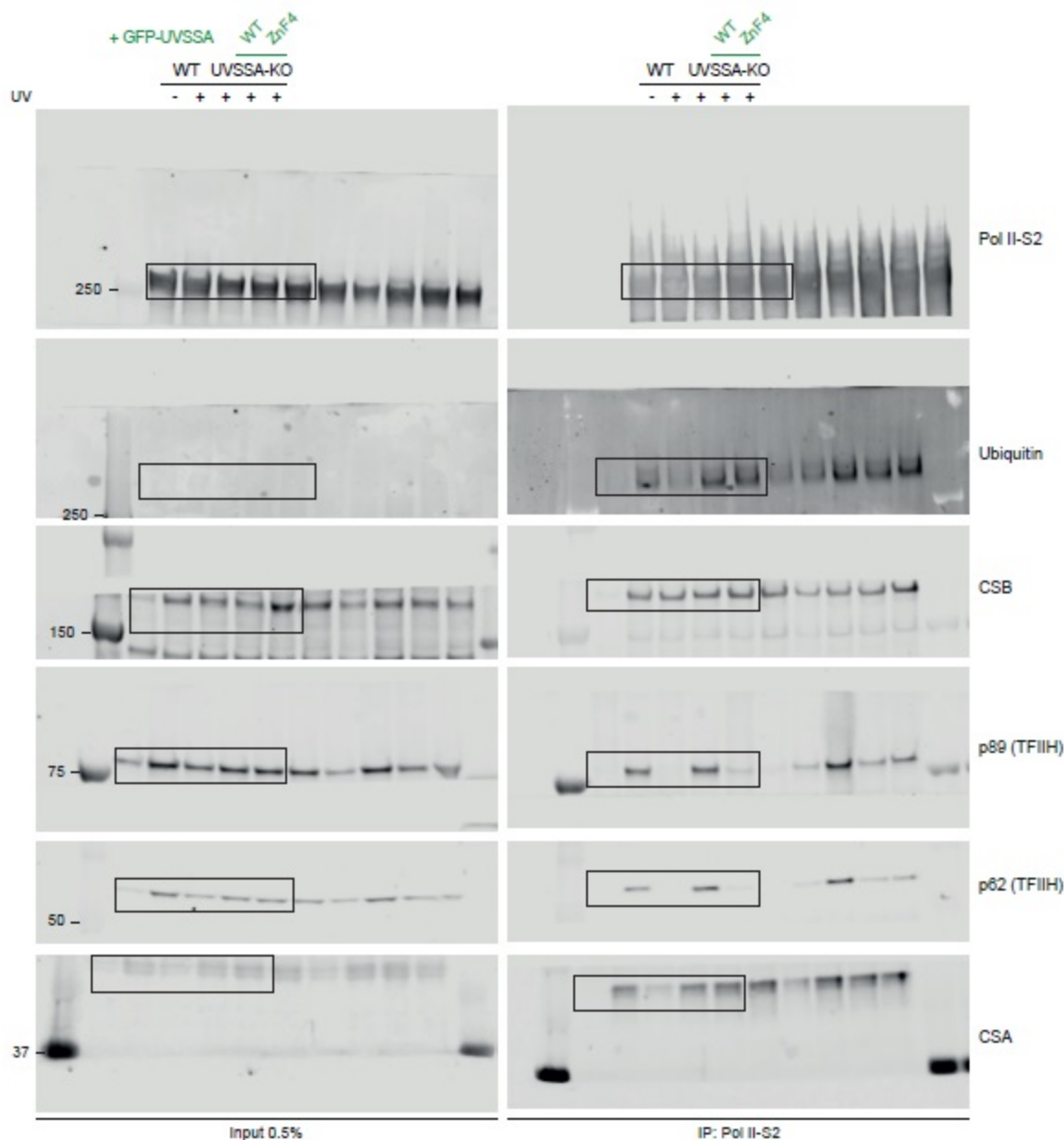

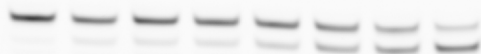

Supplement: Supplementary file 15 — Unprocessed western blots. [file 41594_2023_1207_MOESM15_ESM.pdf]

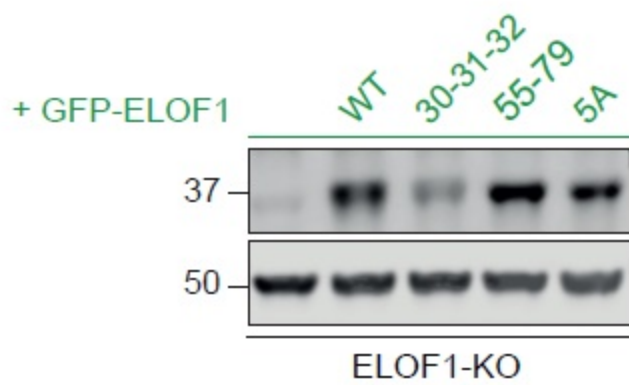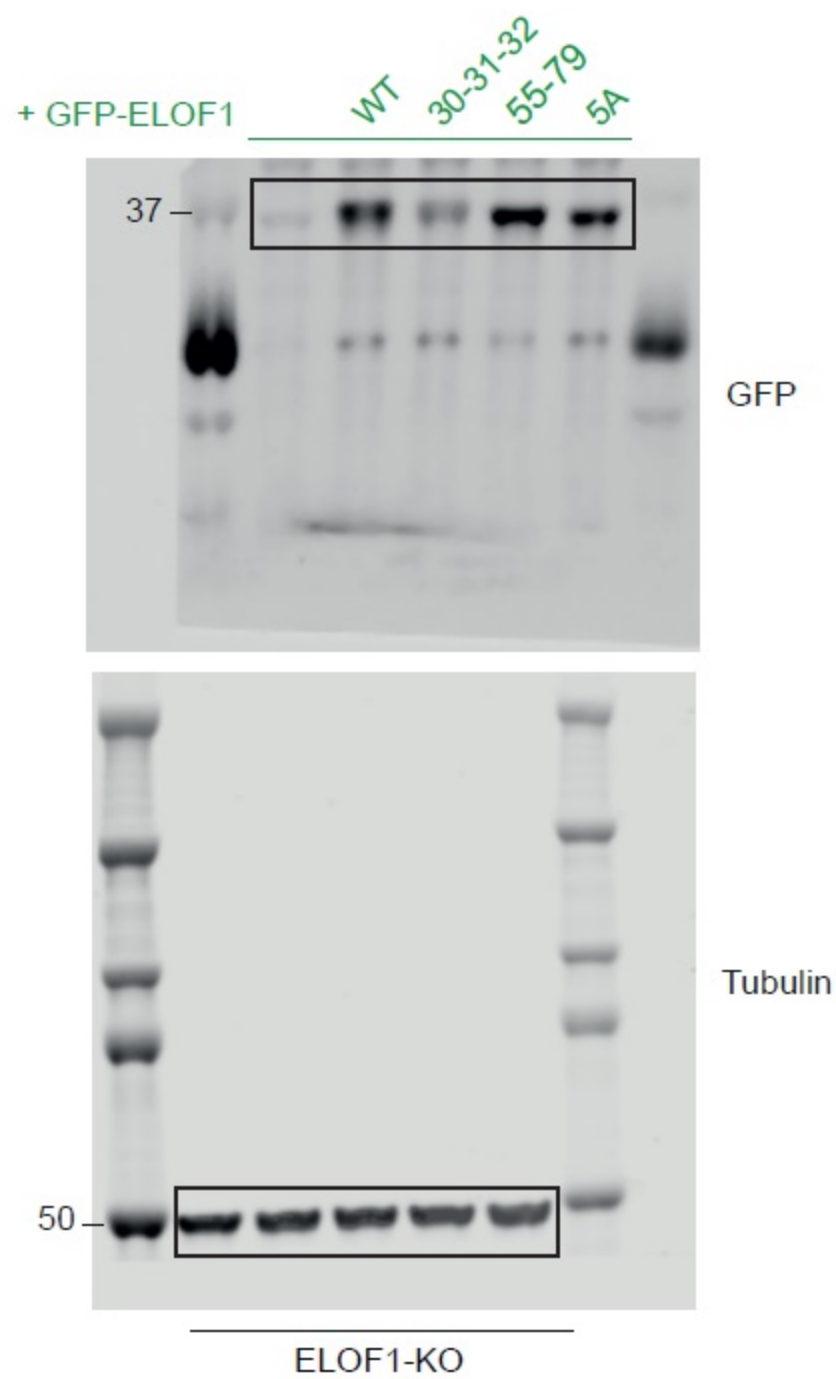

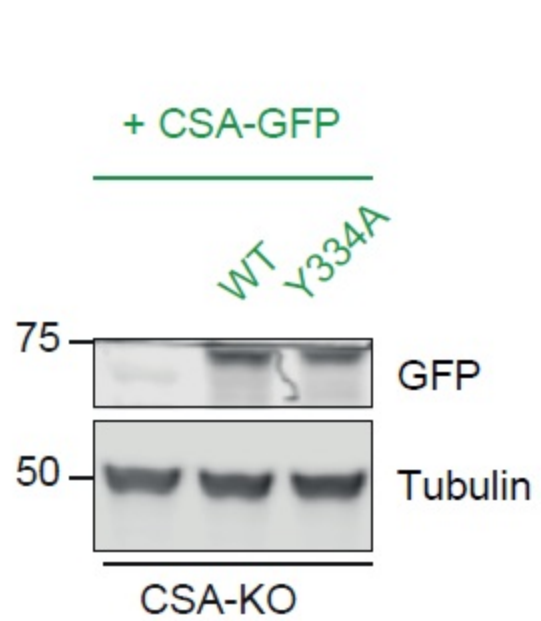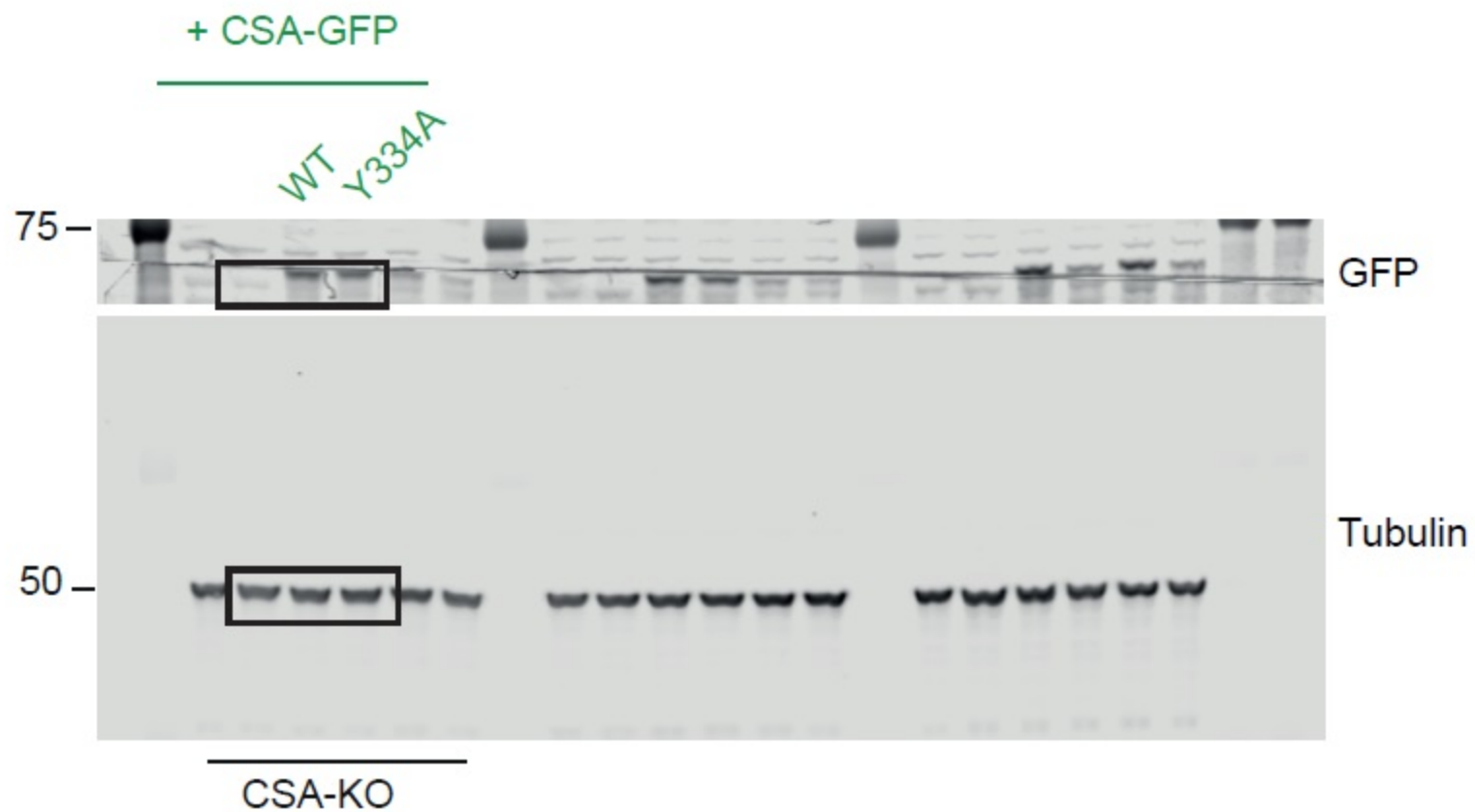

Supplement: Supplementary file 16 — Unprocessed western blots. [file 41594_2023_1207_MOESM16_ESM.pdf]

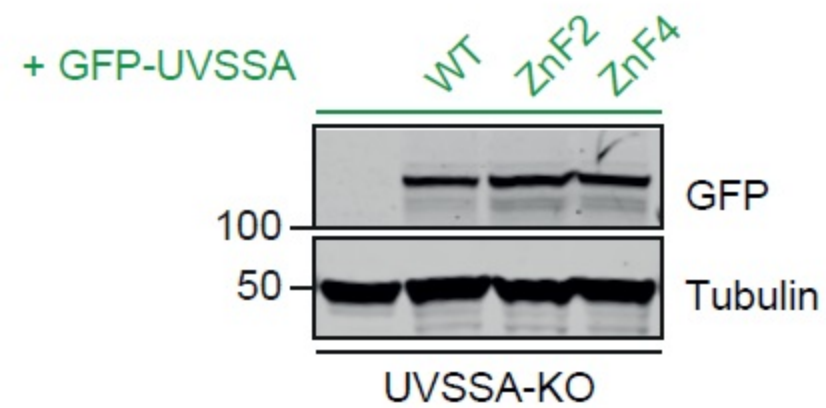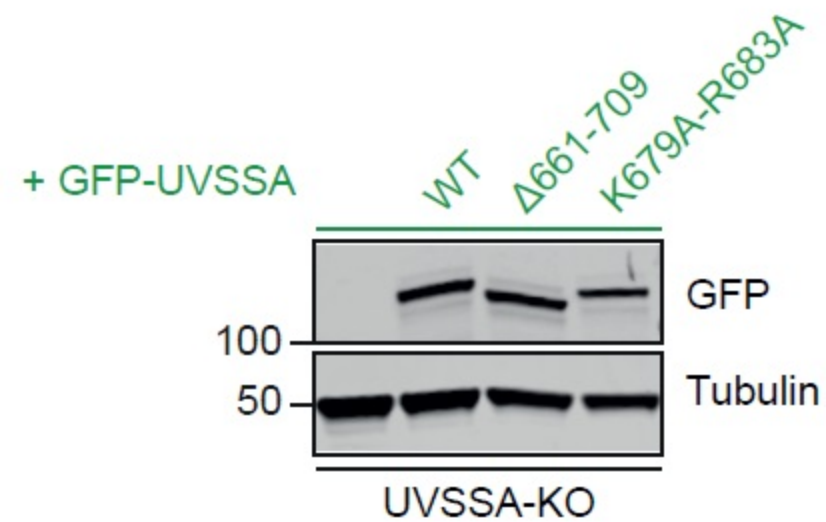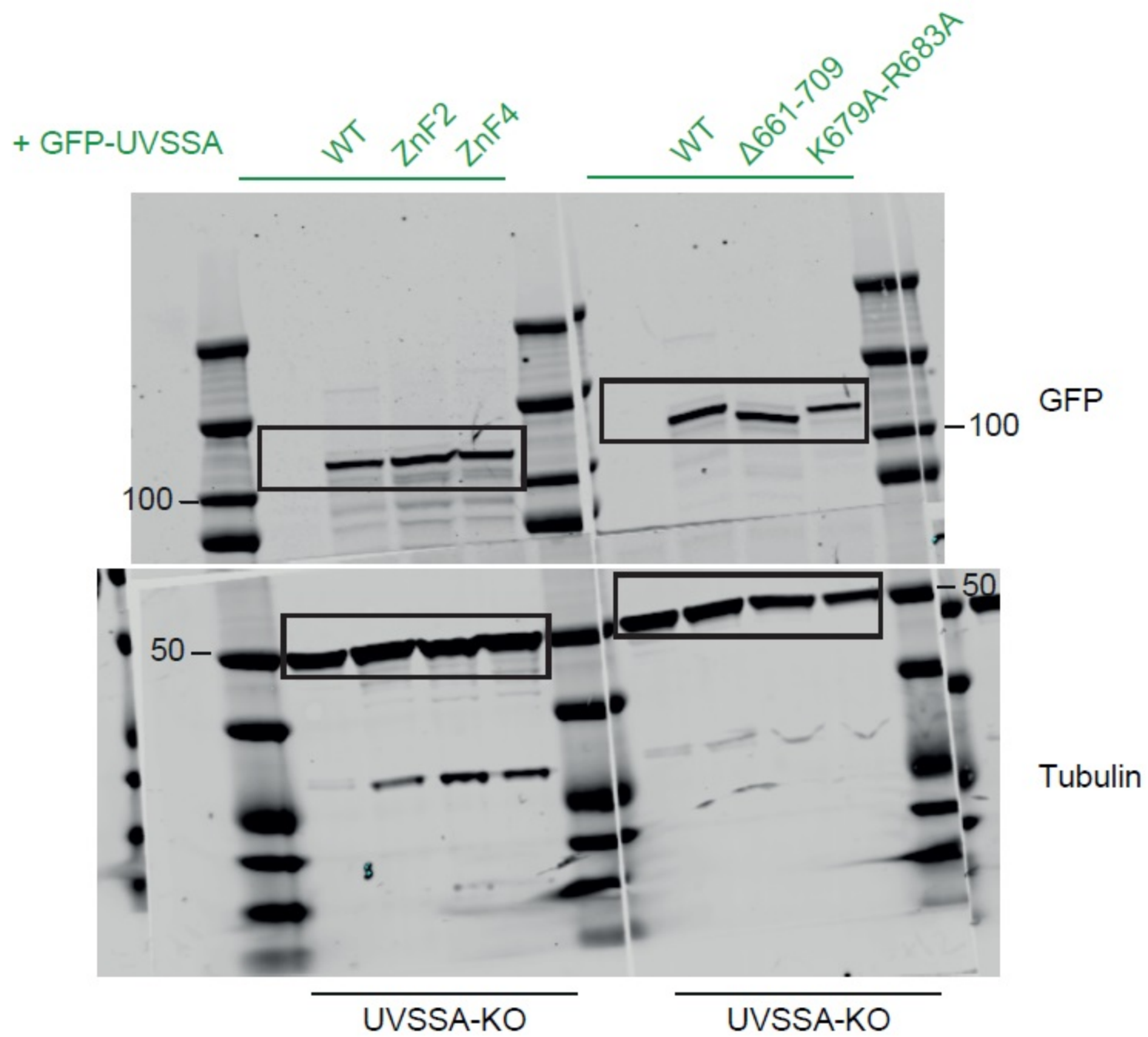

Supplement: Supplementary file 19 — Unprocessed western blots. [file 41594_2023_1207_MOESM19_ESM.pdf]

WT ZnF4 Δ661-709 K679A-R683A  
WT

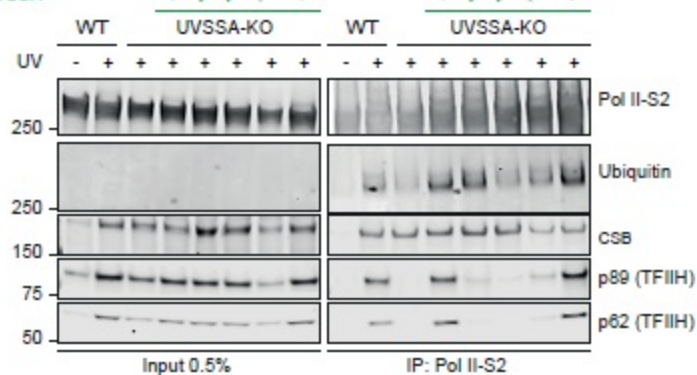

+ GFP-UVSSA

|    | WT |   | UVSSA-KO |   |   |   |   |   |
|----|----|---|----------|---|---|---|---|---|
| UV | -  | + | +        | + | + | + | + | + |

UV

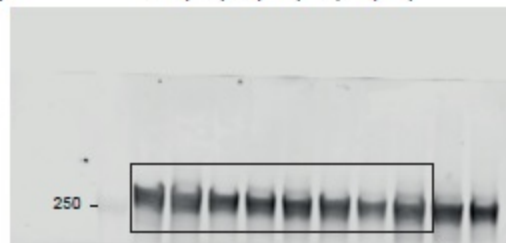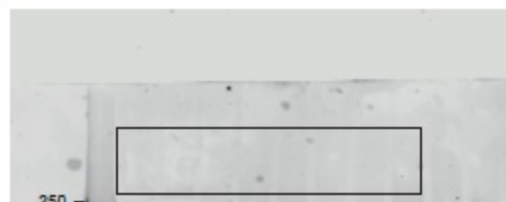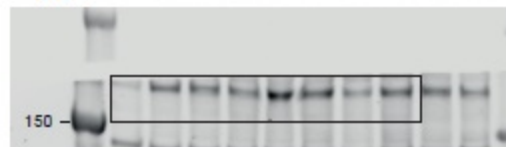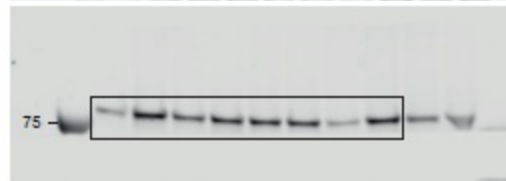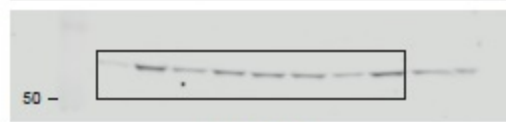

Input 0.5%

| WT |   | UVSSA-KO |      |         |             |    |   |
|----|---|----------|------|---------|-------------|----|---|
| -  | + | -        | +    | +       | +           | +  | + |
|    |   | WT       | ZnF4 | Δ61-709 | K679A-R683A | WT |   |

—

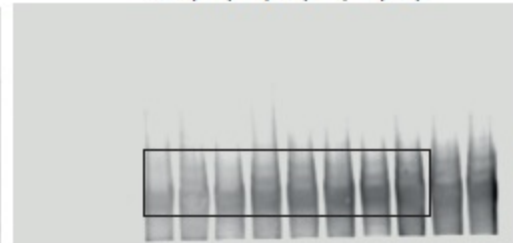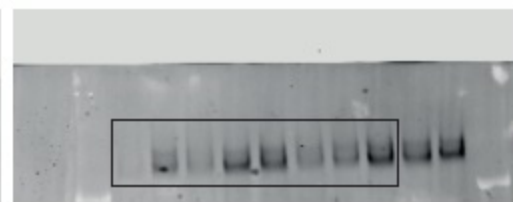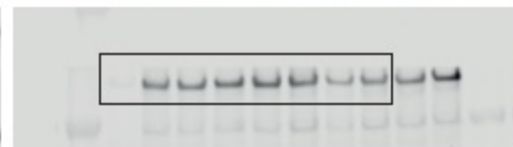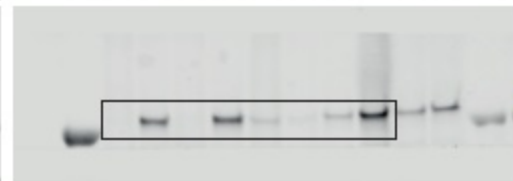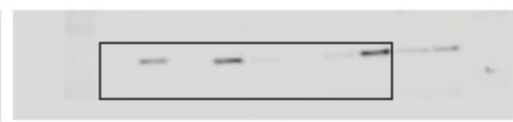

IP: Pol II-S2

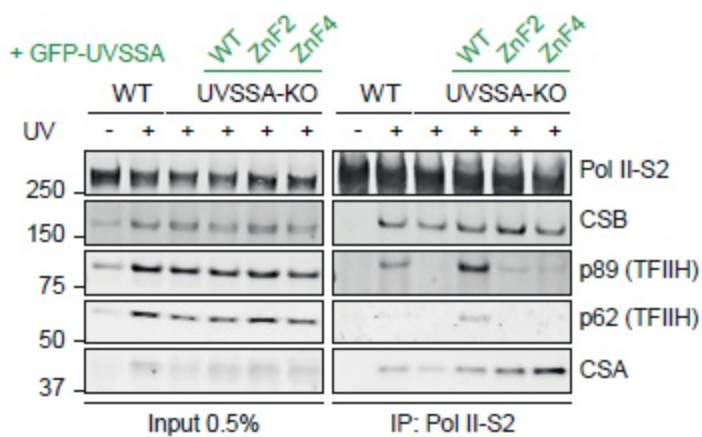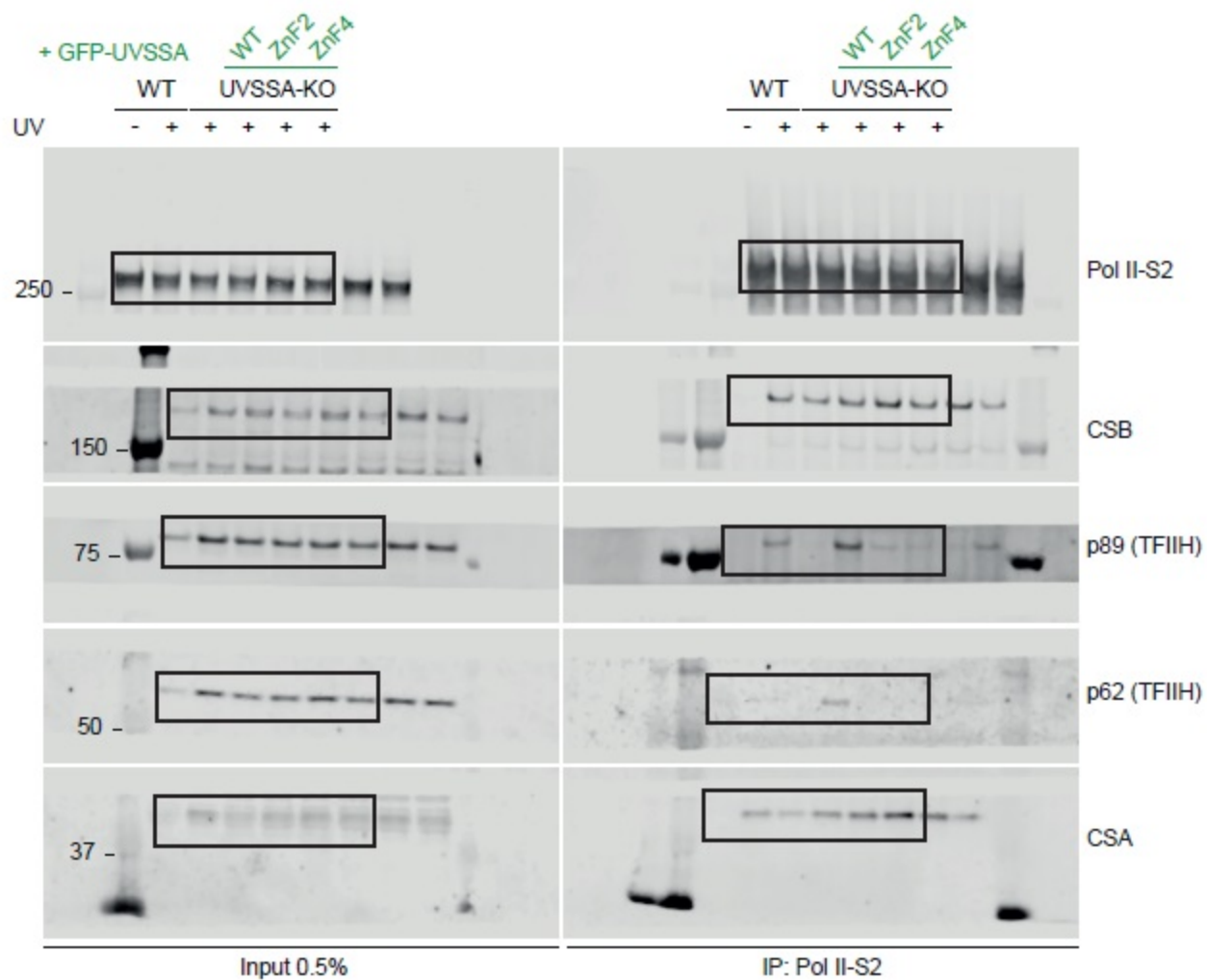

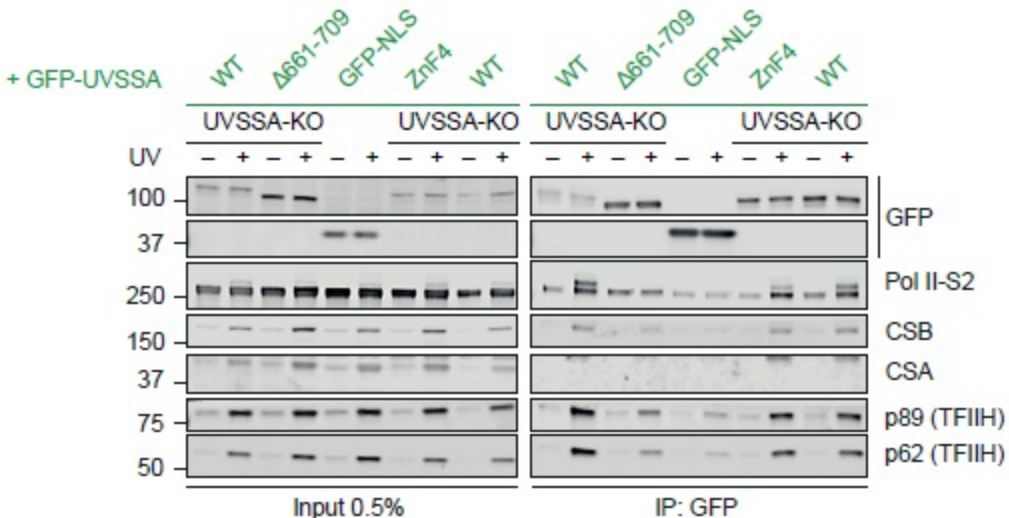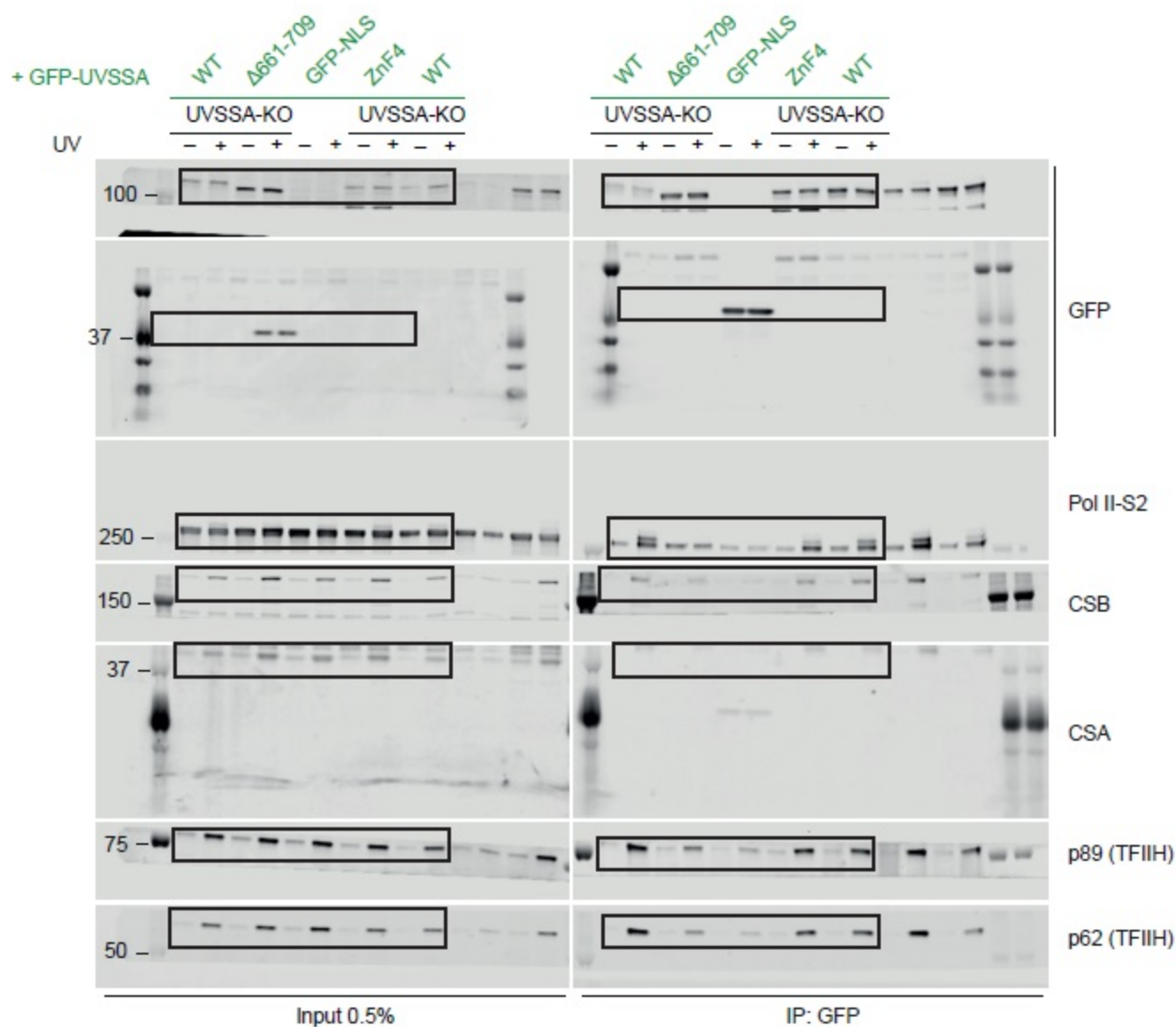

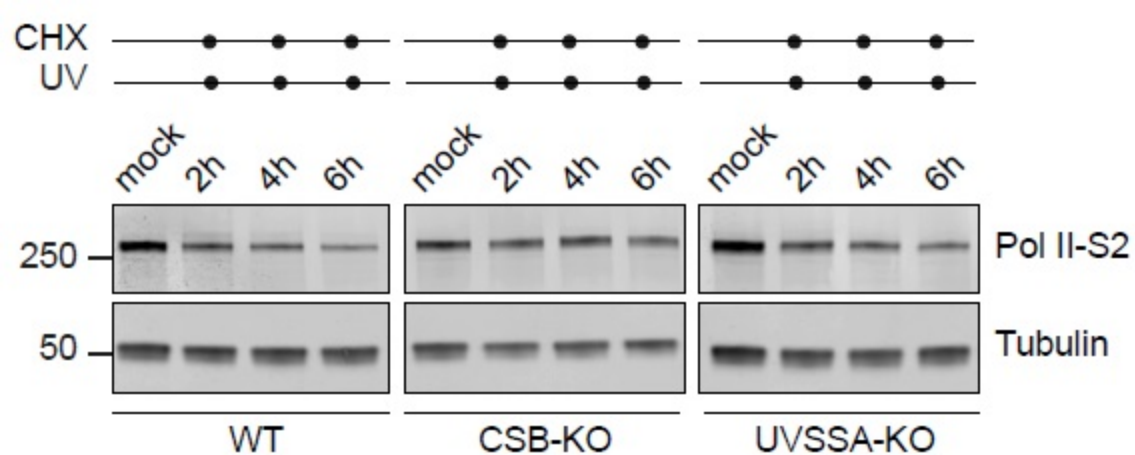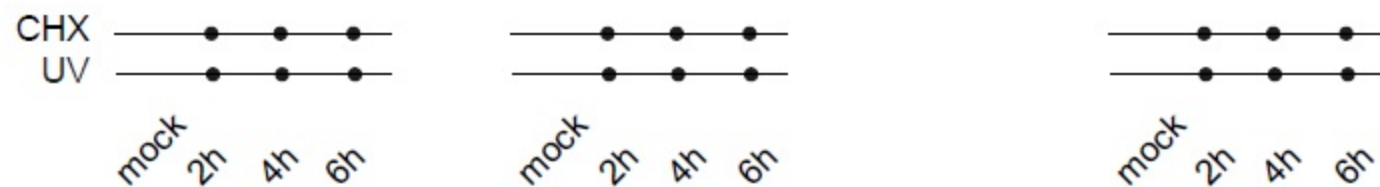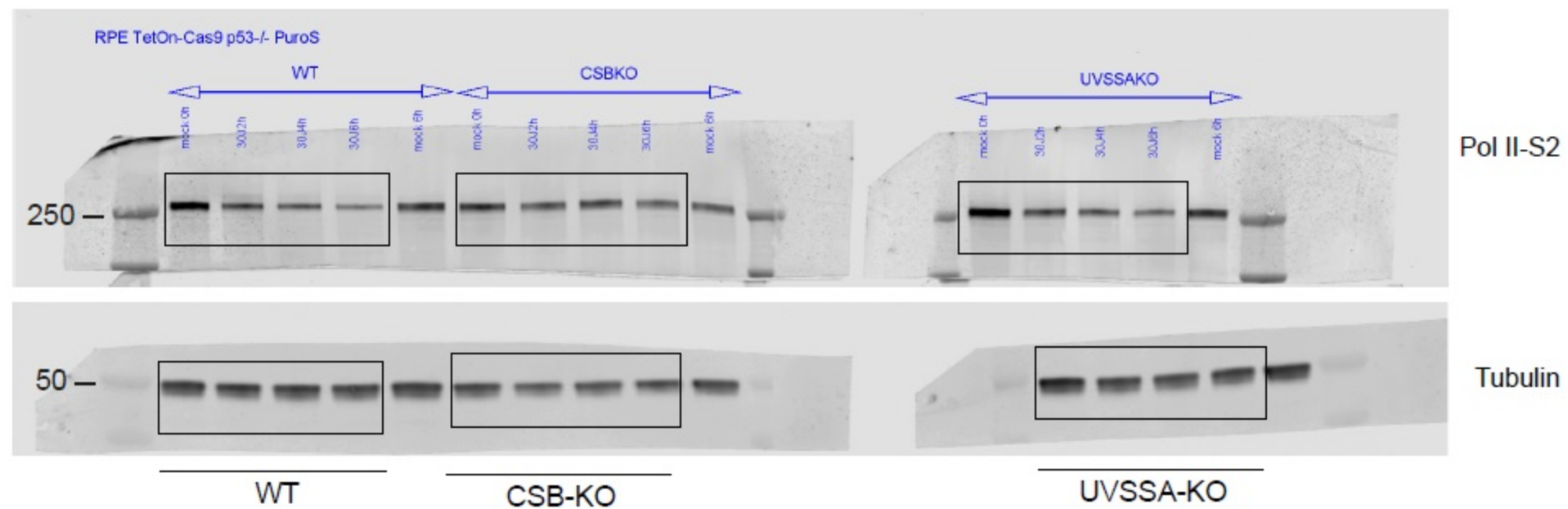

Supplement: Supplementary file 20 — Unprocessed western blots. [file 41594_2023_1207_MOESM20_ESM.pdf]
